# Synthesis of enhanced phosphonic functional groups mesoporous silica for uranium selective adsorption from aqueous solutions

**DOI:** 10.1038/s41598-017-11993-5

**Published:** 2017-09-15

**Authors:** H. Sarafraz, A. Minuchehr, Gh. Alahyarizadeh, Z. Rahimi

**Affiliations:** Engineering Department, Shahid Beheshti University, G.C., P.O. Box 1983969411, Tehran, Iran

## Abstract

Enhanced phosphonic functional group (PFG)-based mesoporous silicas (MSs) were synthesized by hydrothermal method for uranium [U(VI)] selective adsorption from aqueous solutions. Considering that PFGs are directly related to U(VI) adsorption, the main idea of this research was to synthesize enhanced PFG-MSs and consequently enhance U(VI) adsorption. We synthesized two kinds of MSs based on acetic and phosphoric acids at weakly acidic pH, which allows high-loading phosphonic functionality. The main sodium and phosphonic functionality sources were sodium metasilicate and diethylphosphatoethyltriethoxysilane (DPTS). Adsorption experiment results exhibit enhanced U(VI) adsorption capacity from 55.75 mg/g to 207.6 mg/g for acetic and phosphoric acids, respectively. This finding was due to the enhancement of PFGs by phosphoric acids. The highest adsorption selectivity was 79.82% for U(VI) among the six different elements, including Pb, As, Cu, Mo, Ni, and K. Structural characterization of the samples was performed by Fourier transform infrared, X-ray diffraction, scanning electron microscopy, energy-dispersive X-ray spectroscopy, and Brunauer–Emmett–Teller analysis methods. Element concentrations were measured by inductively coupled plasma optical emission spectrometry. Several parameters affecting adsorption capacity, including pH, contact time, initial U(VI) concentration and solution volume, and adsorbent concentration, were also investigated.

## Introduction

The specific and fundamental properties of the materials and methods used for the removal and recovery of radionuclides from the aqueous wastes, which have attracted significant interest, are highly efficient and selective separation. Uranium as one of the radionuclides and the major fuel for nuclear reactors exhibits serious threat to the environment through most processes and activities related to nuclear fuel cycles including mining, milling, and spent nuclear reprocessing. The separation and recovery of uranium display great significance from two points of view, namely, removal for environmental protection and repossession for utilization of uranium resources. Uranium exhibits specific properties including a long half-life radionuclide and a complicated coordination chemistry. It can be found in several stable oxidation states especially at four and six in the solid and aqueous forms. The hexavalent form of uranium, namely, U(VI) as the mobile and aqueous uranyl ion (UO_2_^2+^), is the typical form of uranium under ordinary environmental conditions^1–3^.

Several methods including solvent extraction^4^, coprecipitation^5^, chemical precipitation^6^, chromatographic extraction^7^, membrane dialysis^8^, flotation^9^, ion exchange^10^, and adsorption^11–15^, have been designated for uranium removal from aqueous solutions. Among the mentioned methods, adsorption is the best and effective candidate for U(VI) removal from aqueous solutions with moderate and low uranium concentrations. Several different kinds of adsorbents have been synthesized, developed, and tested for uranium adsorption from aqueous solutions, e.g., organosilicon^12^, polyurethane foams^11, 13^, polyacrylonitrile^15^, polypropylene fiber^11^, and imprinted polymers^14^. Four main characters including efficiency, selectivity, availability, and cost effectiveness are the comparative factors, which define the usefulness of these adsorbents.

One of the most promising materials, which are used for ion separation applications in environmental remediation, metallurgical purposes, and nuclear engineering, are ordered mesoporous silicas (MSs). MSs are already widely used in different applications including catalysis^16^, drug delivery, sensing^17^, and optics. To date, several MSs have been synthesized as the solid ligands mainly to extract and remove heavy metal cations from aqueous solutions and wastewaters. Phosphonic acid derivatives involved in porous silicas as the functional groups, which are the main reason for enhanced extraction and separation efficiency of rare-earth metals and actinides^18^, are of great interest for sorption technologies. Information about the properties of active sites, adsorption capacity, and accessibility for sorbates can be achieved by studying MS functional groups^19^. Sol–gel^20^, surface modification^21^, and template^22^ methods are the most common methods to synthesize MSs. The sol-gel method is a commonly used technique to synthesize of silica nanoparticles. This process involves the growth of networks which is called sol. The sol is prepared by an arrangement of the colloidal suspension. The sol-gel process will be finalized by gelation step to form a system in continuous liquid phase which is called gel. The sol-gel method has different advantages and disadvantages. The main disadvantage of the sol-gel process is the long processing times and the high cost of raw materials. Other sol-gel disadvantages are very large shrinkage of the gel products in the process of gel drying, the presence of undesirable residuals like hydroxyls and organics^20, 23, 24^. One of the important step to prepare and enhance the silica-polymer nanocomposites properties is the chemical modification of silica surface. The affinity between the organic and inorganic phases of the silica-polymer nanocomposites are enhanced by the surface modifications. The surface modification is the most effective technique for coupling the silane agents to the structure and can improve the dispersion of silica nanoparticles within the polymer matrix^25, 26^. The template method is another technique to synthesize the silica nanoparticles which widely used in recent years. This method is often categorized into two classifications; hard and soft template methods. Due to the significant advantages of the soft template including simplicity of the process, and good repeatability, this method has widely used for development in the synthesis of nanomaterials^26^. Production method effectively affects the adsorption properties of the MSs.

G. R. Yurchenko *et al*. reported the adsorption properties of MSs containing phosphonic acid residues synthesized by combining the sol-gel and the template method. They showed that all of the synthesized samples possess high-specific surface areas. They also indicated that the accessibility of active acidic sites, which determine the adsorption capacity and is related to the interaction between the active sites and electron-donor molecules, depends on the surface layers and pore structures^27^. Applying a specific template (P123) in this work due to the simplicity of the process, and high content of functional groups can be proposed in the future works. Oksana A. Dudarko *et al*. prepared the MSs with phosphonic functional groups under conventional and microwave condition. They used tetraethyl orthosilicate as a component framework and DPTS agent as a source of functional groups. They investigated several parameters, such as certain physical level, pore size, and pore volume. Their results showed slight difference in the surface area of MSs between two methods^28^. Although in the microwave condition, the synthesis time is shorter than conventional condition, a lower specific surface area is obtained in this method. The same nanoparticle structure is also obtained in the two mentioned methods^28^. A novel phosphonic acid-functionalized silica magnetic sorbent (PA-SMM) was prepared by Limin Zhou *et al*. to remove U(VI) from aqueous solution. They showed that the PA-SMM can be regenerated by using eluent involving ethylenediaminetetraacetic acid and HNO3^29^. They applied the surface modification to add the phosphonic functional group to the magnetite nanoparticle to enhance the U adsorption. DPTS is the main source of the phosphonic functional group in this process. Oksana A. Dudarko *et al*. synthesized different MSs with various molar ratios of the sodium metasilicate and DPTS. They reported the highest uranium adsorption capacity of 54.5 mg/g for 10:2 molar ratio^30^.

In this study, a novel MS with enhanced phosphonic functional groups was synthesized by hydrothermal method for uranium [U(VI)] selective adsorption from aqueous solutions. Two kinds of MSs based on acetic and phosphoric acids at weakly acidic pH, which allows high loading phosphonic functionality, were synthesized. Several parameters, which influence the adsorption capacity including pH, contact time, initial U(VI) concentration and solution volume, and adsorbent concentration, were also investigated. Regarding the advantage of P123 as a template and DPTS as a source of the phosphonic functional group, the soft template and surface modification were selected as the main procedure in this work. Compared to previously reported studies, several improvements were expected including 1) enhanced phosphonic functional group which results in heightened uranium adsorption, 2) selective adsorption of uranium, 3) highly reduced equilibrium adsorption time, 4) shortened synthesis time, and 5) no requirement to the vacuum environment in the drying process.

## Experimental section

### Chemicals

Sodium metasilicate, Na_2_SiO_3_·9H_2_O (Sigma, USA), diethylphosphatoethyltriethoxysilane, (C_2_H_5_O)_3_Si(CH_2_)_2_P(O)(OC_2_H_5_)_2_ (DPTS, 95%, Gelest, USA), poly(ethylene oxide)-poly(propyleneoxide)-poly(ethylene oxide), Pluronic P123 block copolymer (EO_20_RO_70_EO_20_, 99%, BASF, USA), concentrated acetic acid, CH_3_COOH (99.7%; Merck, Germany); *ortho*-phosphoric acid (85%; Merck, Germany), ethanol (absolute), and uranyl nitrate [UO_2_(NO_3_)_2_·6H_2_O; Aldrich, USA] were used in the synthesis and uranium adsorption of P-containing MSs.

### Procedures of P-containing mesoporous silica samples

**Figure 1:**
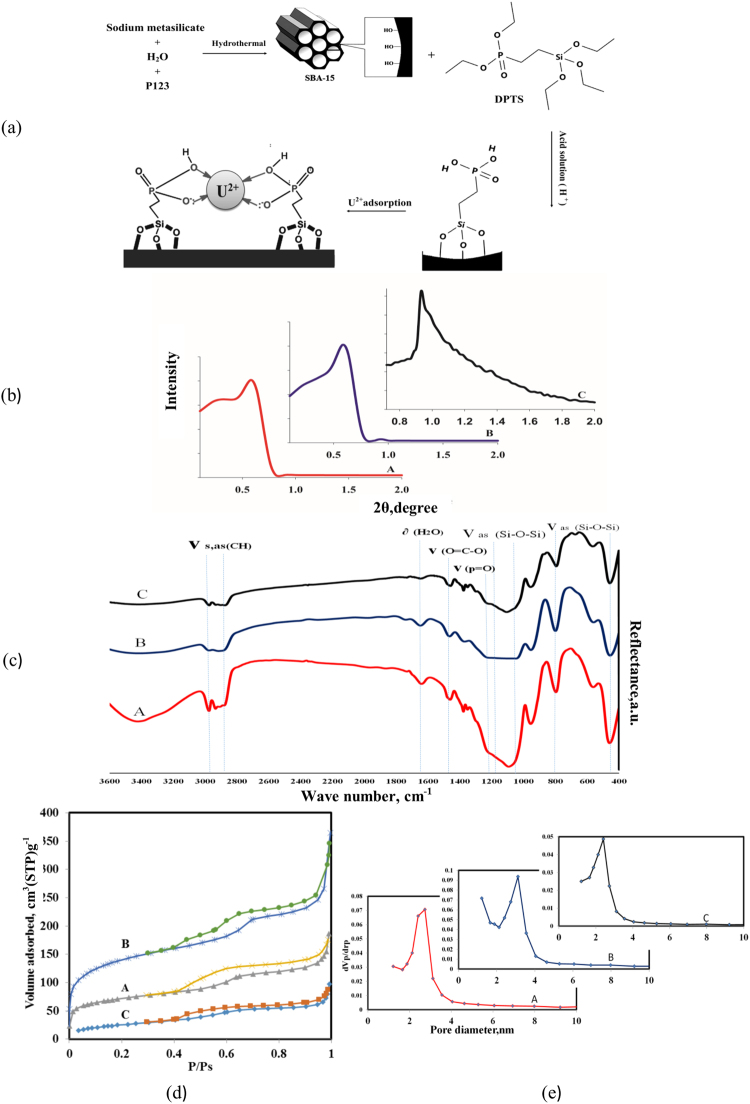
(**a**) The schematic of the formation mechanism of phosphonic functional groups mesoporous silica. The structural characteristics of the acetic acid based synthesized MSs including (**b**) XRD patterns, (**c**) FT-IR spectra, (**d**) BET surface areas, and (**e**) pore size distributions.

The two kinds of MSs were prepared based on using acetic and phosphoric acids. The acetic-based MSs were obtained by using the Na_2_SiO_3_·9H_2_O/DPTS molar ratio = 10:1 (2 and 3). First, 0.25 cm^3^ of DPTS was mixed with 7.36 cm^3^ of concentrated acetic acid. The mixture was prehydrolyzed at 80 °C for 6 h on the oil bath. Subsequently, a solution containing 0.8 g of Pluronic P123 in 13 cm^3^ water was added to the room temperature-cooled DPTS mixture. The final mixture was prepared by adding a solution containing 2.276 g (0.008 M) of sodium silicate dissolved in 10 cm^3^ water. Then, the final mixture, which is a kind of suspension, was left in the oil bath for 1 h at 40 °C. Finally, the resulting mixture was placed in the conventional oven for 2 h at 40 °C with magnetic stirring and 10 h at 100 °C without stirring^30^. The subsequent process included the treatments to remove the template. Up to 2.5 g of synthesized MS was boiled in the mixture of methanol and HCl. The amount of used methanol and HCl with respect to MS was 30:10:1 mass ratio. The template removal treatment was replicated thrice^31^. The final MS sample was filtered, dried for 8 h under 400 °C and atmospheric pressure in the furnace. The enhanced phosphonic functional group (PFG) MS was prepared based on using phosphoric acids with the Na_2_SiO_3_·9H_2_O/DPTS molar ratio = 10:1 (2 and 3). The schematic of the formation mechanism of phosphonic functional groups mesoporous silica is shown in Fig. 1(a). First, the host template was fabricated by hydrolyzing mixture of the template (P123) and silica source in the water and acid environment. Then the alkoxy groups of silane which prepared by hydrolyzed DPTS in the acid solution was added to the silica structure to form phosphonic functional groups which is called surface modification^1, 27, 32^.

#### Characterization

Structural characterization of the synthesized MSs was established by Fourier transform infrared (FT-IR), X-ray diffraction (XRD), scanning electron microscopy (SEM), energy-dispersive X-ray spectroscopy (EDS), and Brunauer–Emmett–Teller (BET) analysis methods. XRD Bruker D8 Advance was used to collect XRD patterns at 30 kV and 20 mA and Cu Kα radiation (λ = 0.1540598 nm). FT-IR spectrometry TENSOR 27 was used to record FT-IR spectra of synthesized samples. The morphology and particle sizes of synthesized MSs were measured by field emission SEM (Zeiss-Sigma-VP-500). The BET surface area and pore size distributions were obtained from the nitrogen adsorption–desorption isotherms obtained by BELSORP-mini analyzers (BEL Japan, Inc.). The element concentrations in adsorption experiments were performed by SPECTRO Genesis simultaneous charge-coupled device-based radially viewed inductively coupled plasma optical emission spectrometry (ICP-OES).

#### Adsorption tests

The uranium and other element adsorption experiments were performed by preparing different volumes and concentrations of aqueous solutions containing all the considered elements with various solution pHs. The element concentrations were estimated before treatment and after 5–60 min. The adsorption capacity and removal percentage (%) of each element were calculated using the following equations, respectively:

1 $${\rm{Adsorption}}\,{\rm{capacity}}({\rm{mg}}/{\rm{g}})=\,\frac{({C}_{i}-{C}_{f})\ast V}{m},$$

2 $${\rm{Removal}}\,( \% )=\frac{({C}_{i}-{C}_{f})\ast 100}{{C}_{i}},$$

where *C*_*i*_ is the initial uranium concentration, and *C*_*f*_ is the uranium concentration at equilibrium after treatment with synthesized MSs. *m* (*mg*) and *V* (*ml*) are the mass of the adsorbent and the volume of the solution, respectively^33^.

## Results and Discussion

The structural characteristics of the acetic acid-based synthesized MSs including the XRD patterns, FT-IR spectra and BET surface areas, and pore size distributions are shown in Fig. 1. As shown in Fig. 1(b), the XRD patterns confirm the presence of ordered mesostructures for the synthesized MSs based on using acetic acid. The ordered mesostructures contain two main well-defined reflections at 2θ, namely, the peak of (1 0 0) at 0.6°–0.8° and the peak of (1 1 0) at 0.9°–1.1°. This condition indicates that the synthesized MSs display the hexagonal structure (the p6m symmetry group)^34^. The main extracted point from the XRD patterns is that the synthesized MS with the Na_2_SiO_3_·9H_2_O/DPTS molar ratio of 10:2 shows more ordered structures than that of others. Figure 1(c) shows the infrared (IR) spectra for the MSs with the three molar ratios of 10:1 (sample A), 10:2 (sample B), and 10:3 (sample C). As shown in this figure, the IR spectra of MSs include several main absorption bands. The first group of absorption bands is related to the ν_as_(Si–O–Si) frequencies located in the 410, 800, and 1060–1160 cm^−1^. The absorption band ν(P=O), which is related to the P-containing groups because of the initial DPTS located at 1241 cm^−1^ on the spectrum. The absorption peak at approximately 1630 cm^−1^ and a broad absorption band above 3000 cm^−1^ are related to the deformation vibrations of H_2_O and stretching vibrations of OH. An absorption peak exists at 1466 cm^−1^, which are characteristic of symmetric stretching vibrations of the O=C–O group. The last part of IR spectrum refers to the ν_s,as_(CH) located at the 2920–2986 cm^−1^ ^35, 36^.The N_2_ sorption–desorption isotherm, which determines the specific surface area and pore size distributions, was measured at −196 °C by BELSORP mini analyzers. The samples were pretreated at 400 °C for 8 h. The surface specific area was calculated by BET method. Barrett–Joyner–Halenda (BJH) method was also used to calculate pore size distribution curve. The pore size was defined as the maximum of the pore size distribution curve. As shown in Fig. 1(d), the adsorption–desorption isotherms indicate type IV isotherms accompanied by type H4 hysteresis loop^37^, showing the MSs with the pores in the micropore region. The pore size distribution (PSD) of the synthesized MSs is shown in Fig. 1(e), indicating that PSD of synthesized MSs decreases by increasing DPTS ratio (Fig. 1e). Table 1 summarizes the adsorption and structural parameters of synthesized MSs (A, B, and C). As shown in Table 1, the pore diameters of samples decrease by increasing DPTS ratio confirmed by Fig. 1(e). Although increasing total pore volume and specific surface area is expected by decreasing pore diameters, the decreased total volume and surface area were observed for sample C with additional Na_2_SiO_3_·9H_2_O/DPTS molar ratio. The N_2_ adsorption–desorption isotherms show the less steep and the elongated hysteresis loop for sample C with the highest content of functional groups, which indicated that this sample involves non-uniform pores and disordered mesopores that are produced due to additional functional groups concentration. One can argue that the sample A and B present higher specific surface area which affected by the contribution of fine mesopores. The results show that appropriate Na_2_SiO_3_·9H_2_O/DPTS molar ratio cause to form fine mesopores and additional Na_2_SiO_3_·9H_2_O/DPTS molar ratio cause to form disordered mesopores. Another parameters which can derive from the N_2_ adsorption–desorption isotherms is the presence of micropore in the synthesized sample. This parameter also affects the specific surface area. The information around the P/P_0_ of 0.01 and higher P/P_0_ indicates the presence of micropores or absence and filled micropores in the synthesized sample. The N_2_ adsorption–desorption isotherms of synthesized samples show the presence of micropores in sample A and B and absence of micropores in sample C^30, 38^. The XRD pattern, which is shown in Fig. 1(b), confirmed the disordering synthesized MSs by increasing DPTS molar ratio.

**Table 1:** The adsorption and structural parameters of the synthesized MSs based on the acetic acid.

sample	Na_2_SiO_3_·9H_2_O/DPTS	SBET, m^2^/g	Vs, cm^3^/g	dm, nm
A	10:1	245	0.21	3.4
B	10:2	516	0.52	3.2
C	10:3	97	0.13	2.4

Uranium adsorption results of synthesized MSs based on the acetic acid are summarized in Table 2. As can be seen from Table 2, in the identical solution volumes and adsorbent mass conditions, sample B shows the highest value of uranium adsorption capacity and uranium recovery percentage. The two important parameters including high-specific surface area and PFGs are the main reasons causing the higher uranium adsorption capacity of sample B than sample A. As mentioned before, using additional DPTS ratio causes disordering synthesized MS (sample C), presenting an irregular structure and consequently decreased specific surface area and uranium adsorption capacity.

**Table 2:** Uranium adsorption results of synthesized MSs based on the acetic acid.

Sample	Adsorbent mass used (mg)	Volume of solution used (mL)	U adsorption capacity (g-U/kg-ads)	% of U adsorbed
10:1(A)	20	50	30.5	24.3
10:2(B)	20	50	55.75	44.6
10:3(C)	20	50	20.5	16.3

**Figure 2:**
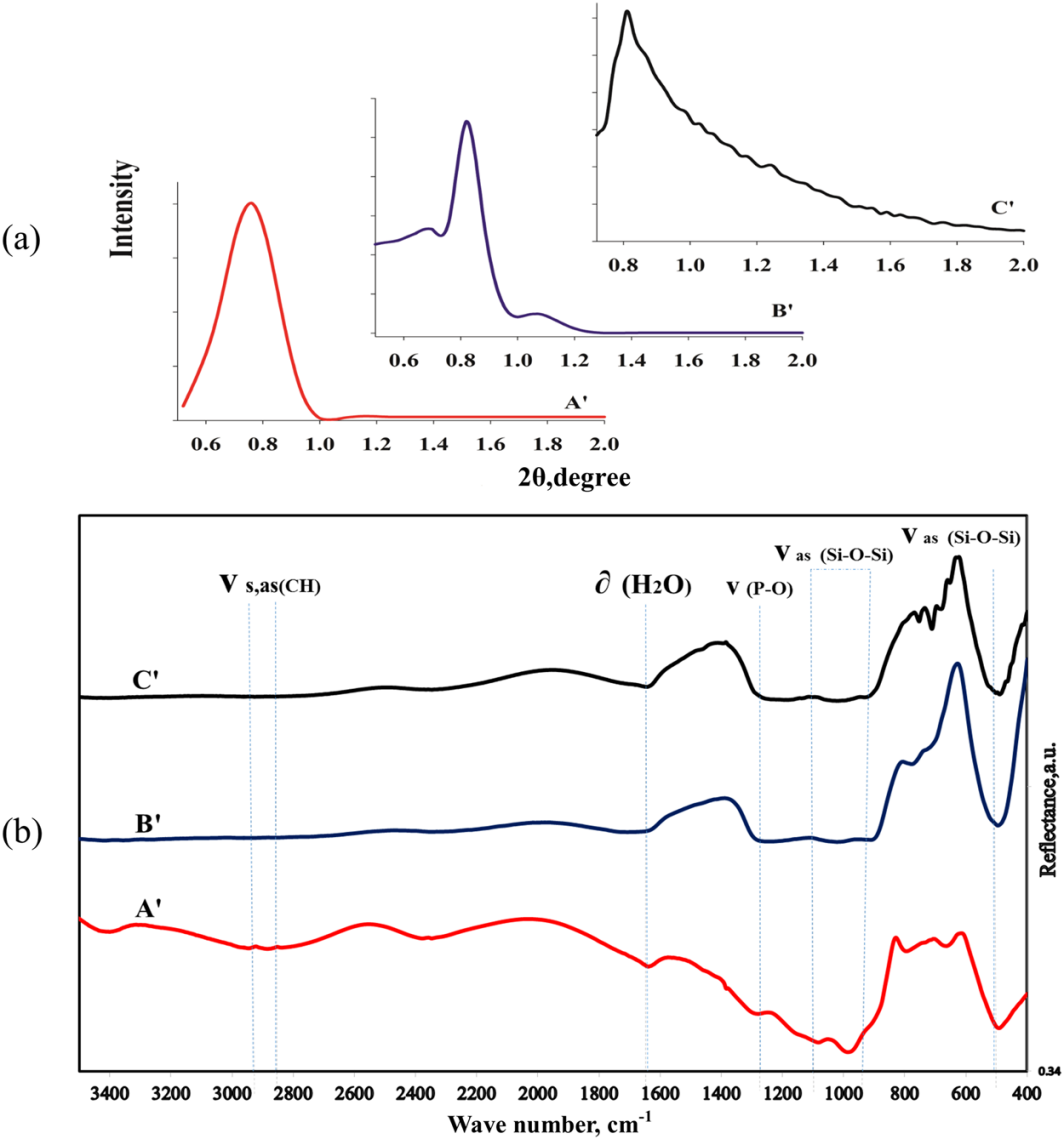
The structural characteristics of the phosphoric acid based synthesized MSs including (**a**) XRD patterns, (**b**) FT-IR spectra.

As mentioned previously, besides the increased specific surface area, one of the foremost factors in the enhanced uranium adsorption is enhanced PFGs without disordering synthesized sample structure. Therefore, the next main goal was finding a new approach to increase PFGs. Instead of increasing DPTS ratio to increase the PFGs, the three new samples (A′, B′, and C′) were synthesized with previous Na_2_SiO_3_·9H_2_O/DPTS molar ratio based on the phosphoric acid instead of acetic acid. Tables 3 and 4 show the uranium adsorption characteristics of the synthesized samples A′, B′, and C′ in the identical conditions and sample B′ in the different adsorption conditions, such as the solution volumes, adsorbent masses, and uranium concentration in the initial solution. As can be seen from Table 3, by using phosphoric acid instead of acetic acid, the uranium adsorption characteristics considerably increased. Enhancing uranium adsorption reaches its maximum value for sample B′ (10:2 molar ratio), which can be due to enhanced PFGs. Table 4 shows the uranium adsorption characteristics at different adsorption conditions, indicating that the uranium adsorption capacity can reach its maximum of 207.595 mg/g. Figure 2 shows the structural characteristics of phosphoric acid-based synthesized MSs. The XRD patterns shown in Fig. 2(a) indicate the synthesized MSs, especially, sample B′ exhibits ordered mesostructures with the hexagonal structure. Figure 2(b) shows the FT-IR spectra of the synthesized MSs based on the phosphoric acid. As shown in this figure, the synthesized MSs of A′, B′, and C′ possess the FT-IR spectra similar to that of A, B, and C MS samples, which include main absorption bands of ν_as_(Si–O–Si), ν(P=O), ν_s,as_(CH), and OH.

**Table 3:** Uranium adsorption results of three synthesized MSs of A′, B′ and C′ based on the phosphoric acid.

sample	Adsorbent mass used (mg)	Volume of solution used (mL)	U adsorption capacity (g-U/kg-ads)	% of U adsorbed
10:1(A′)	20	50	32.75	26.1
10:2(B′)	20	50	90	90
10:3(C′)	20	50	73	58.2

**Table 4:** Uranium adsorption results of synthesized MS of B′ at different adsorption conditions.

Adsorbent mass used (mg)	Volume of solution used (mL)	U concentration of solution (ppm)	U adsorption capacity (g-U/kg-ads)	% of U adsorbed
5	25	42.8	207.595	97.05
10	50	46.8	163	69.7
20	100	46.8	146	62.4
20	50	92.5	132.8	57.4

**Figure 3:**
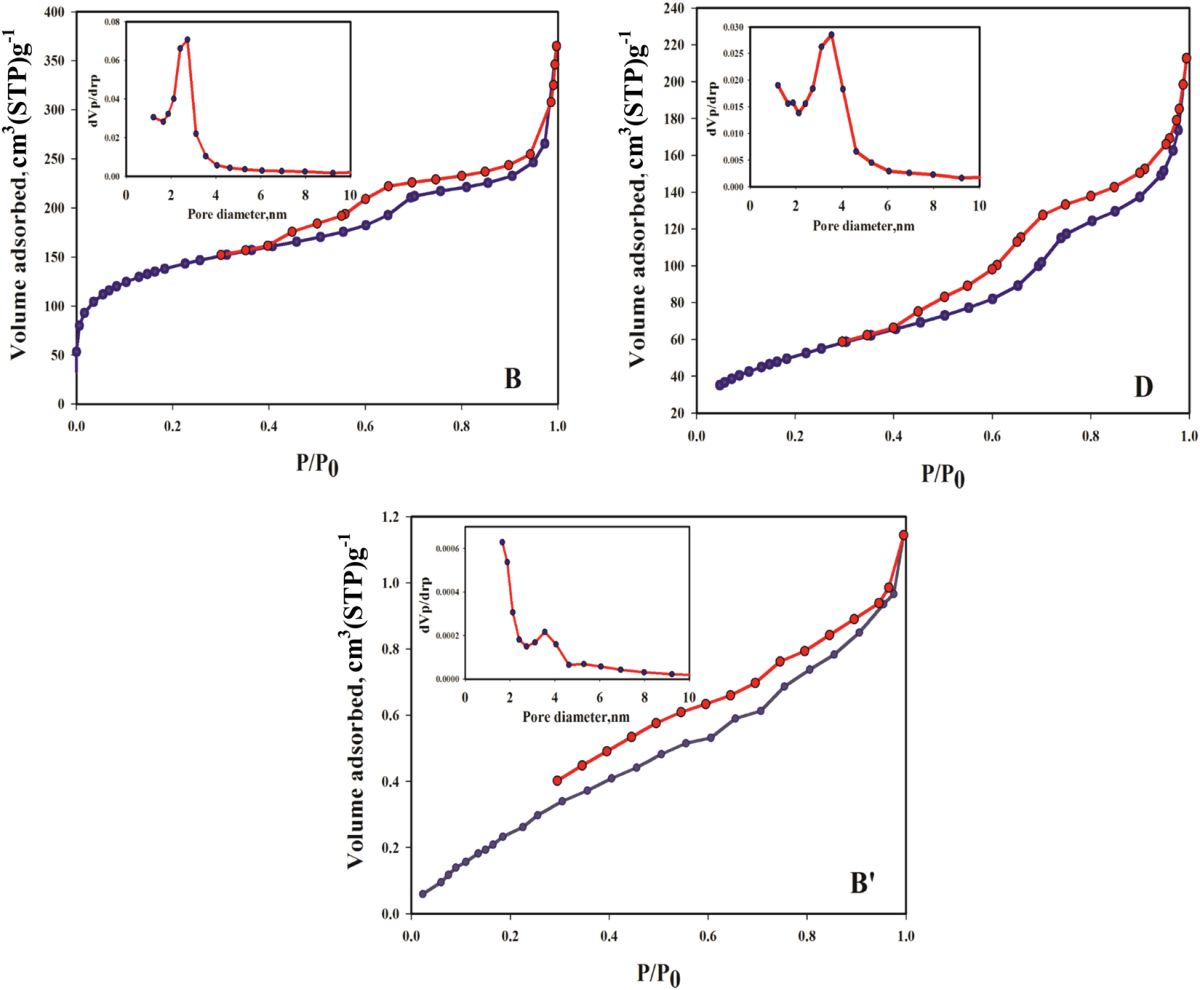
The N2 adsorption–desorption isotherms of the phosphoric acid based synthesized MSs including BET surface areas and pore size distributions.

**Figure 4:**
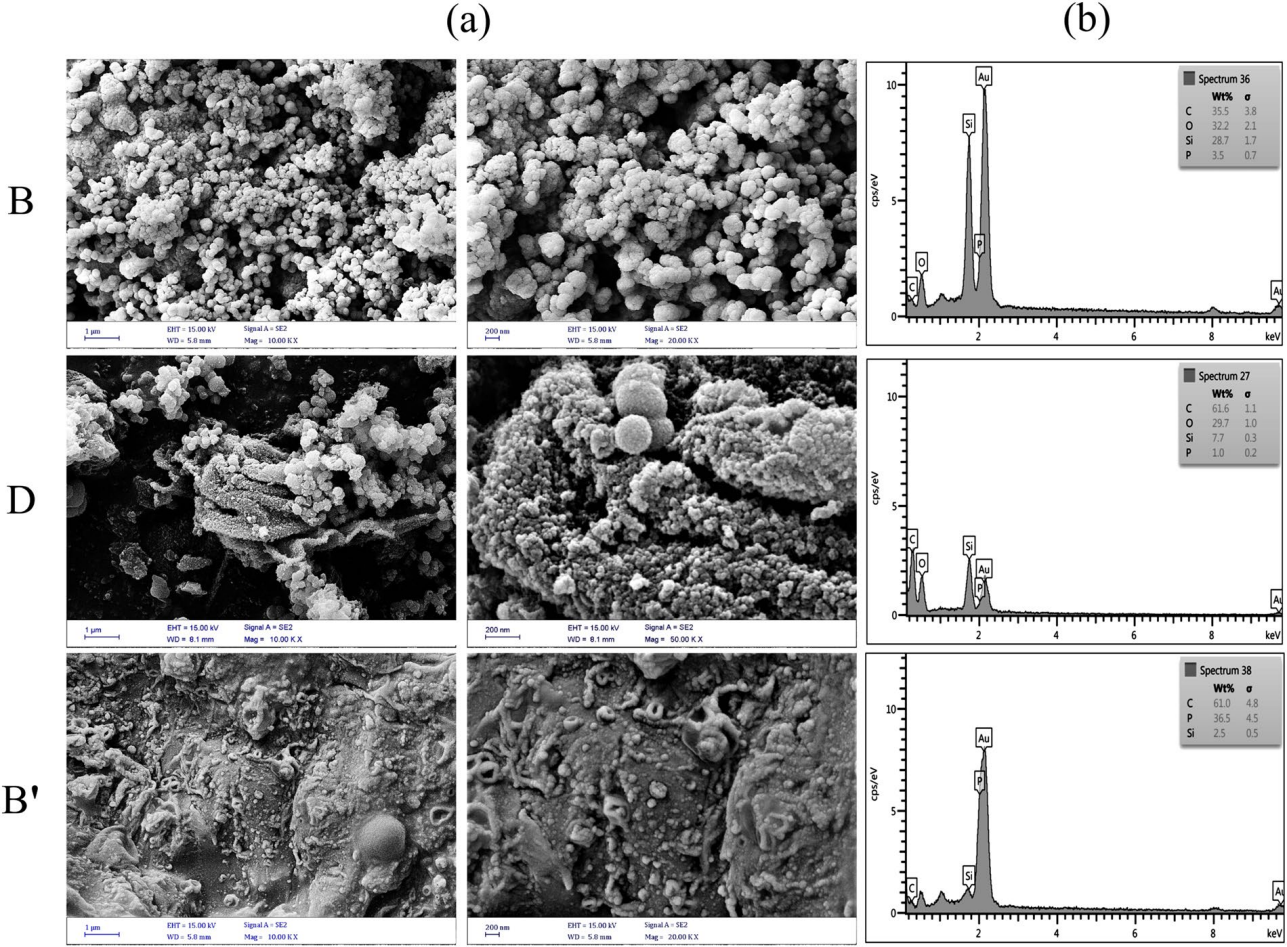
(**a**) SEM images and (**b**) EDS diagrams of the synthesized MSs B, D, and B′.

The surprising part of the synthesis of MSs based on the phosphoric acid is their extremely reduced specific surface area with respect to the acetic acid-based MSs. Figure 3 shows the N_2_ adsorption–desorption isotherms of the phosphoric acid-based synthesized MSs. As shown in these figures, by increasing the phosphoric/acetic acid mass ratios [e.g., 0:100 (B), 3:97 (D); 100:0(B′)], the specific surface area enormously decreased, whereas their N_2_ adsorption–desorption isotherms are still approximately identical, type IV isotherms accompanied by type H4 hysteresis loop. The adsorption and structural parameters of synthesized MSs (B, D, and B′) including specific surface areas, pore diameters, and pore volumes are summarized in Table 5. The reduction in the specific surface area can be due to using phosphoric acid in the MS synthesis. In Fig B′ inside Fig. 3, it may be noted that the N_2_ adsorption - desorption isotherms have not been enclosed, which can be indicated the mesoporous structures extremely reduced. It seems to be due to forming very narrow slit pores or bottle shaped pores which can be located in the micropore structures. Since the N_2_ molecules are very slowly moved at 77 K, their adsorptions are kinetically limited in very narrow pores^39, 40^. Figure 4(a) and (b) show the SEM images and the EDS diagrams of the synthesized MSs B, D, and B′, respectively. The SEM images indicated that by the increasing phosphoric acid mass ratio, the morphologies of synthesized MSs extremely changed, which can be the main reason for specific surface area reduction. As mentioned previously, by increasing the phosphoric acid mass ratio, the phosphonic functional groups were enhanced. Figure 4(b) shows the EDS analysis of synthesized MSs, exhibiting enhanced phosphorus and consequently the enhanced phosphonic functional groups and uranium adsorption. Table 6 shows the uranium adsorption results for different synthesized MSs including 0, 3, 10, 20, and 100 phosphoric/acetic acid mass ratio in the identical solution volumes and adsorbent mass conditions. As shown in this table, the uranium adsorption capacity and uranium recovery percentage increases by increasing phosphoric acid mass ratio, which can be due to the enhanced phosphonic functional groups while reducing specific surface area. The increasing of the phosphonic functional groups can be related to branching out of each existing phosphonic functional groups and producing chelating groups for selective uranium adsorption. A comparison between the maximum adsorption capacities of synthesized MS sample with various previous reported sorbents for uranium adsorption was listed in Table 7. As shown in this table, the synthesized MS sample B′ considerably exhibits a real potential in the uranium adsorption from aqueous solution.

**Table 5:** The adsorption and structural parameters of synthesized MSs (B, D and B′).

sample	Phosphoric acid %	S_BET_, m^2^/g	Vs, cm^3^/g	dm, nm
B	0	516	0.52	4.0
D	3	189	0.32	6.6
B′	100	1.5	0.002	4.6

**Table 6:** Uranium adsorption results for different phosphoric/acetic acid mass ratio.

% of Pho. acid	Adsorbent mass used (mg)	solution Volume (mL)	U adsorption capacity (g-U/kg-ads)	% of U adsorbed
0	5	25	54.5	21.8
3	5	25	59.8	24.5
10	5	25	63.2	27.1
20	5	25	70.7	28.7
100	5	25	207.595	97.05

**Table 7:** A comparison between the maximum adsorption capacities of synthesized MS sample with various previous reported sorbents for uranium adsorption.

Sorbents (ref.)	Adsorption capacity (mg/g)	Sorbents (ref.)	Adsorption capacity (mg/g)
activated carbon^41^	10.47	PA-SMM^29^	76.9
multiwalled carbon nanotubes^42^	24.9	Phosphoric acid modified MS material^43^	66.7
activated charcoal^44^	28.8	Sal-APS-FMNPs^45^	49.0
Fe_3_O_4_@SiO_2_^46^	52.4	PTFG-4^47^	140.68
GO^48^	97.5	Ni0.6Fe_2_.4O_4_ particles^49^	189.04
chitosan grafted MWCNT’s^50^	39.2	PFG MSs (Present study)	207.595
NH_3_-GO^51^	80.13		

**Figure 5:**
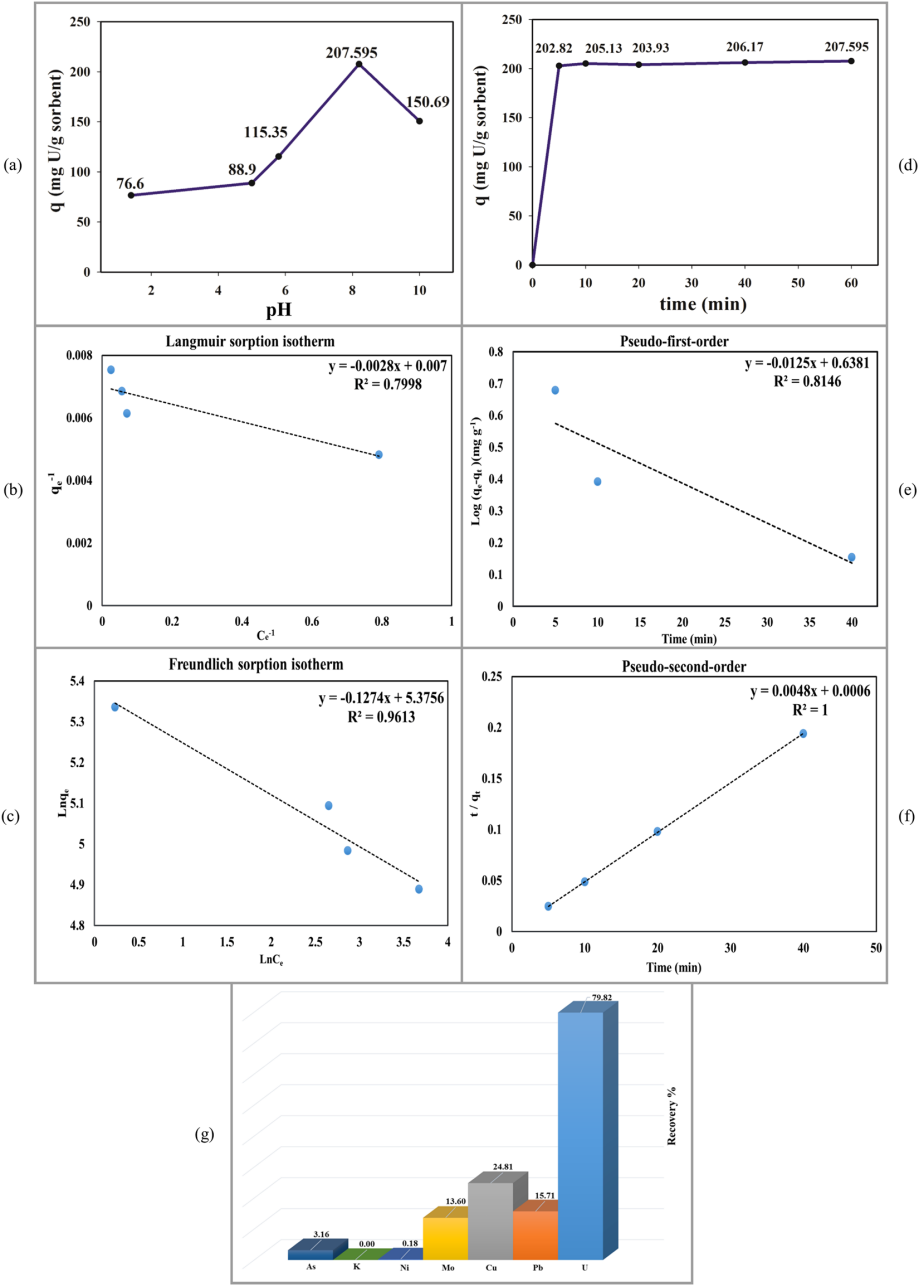
(**a**) The effects of solution pH, (**b**) the Langmuir and (**c**) Freundlich adsorption isotherms, (**d**) the effect of adsorption contact time, (**e**) The pseudo-first- and (**f**) second order kinetic models for uranium adsorption on synthesized MS sample of B′, and (**g**) the effect of interfering ions on the uranium recovery percentages.

The effects of various effective parameters, which influence the uranium adsorption capacity and uranium recovery percentages, were also investigated. All the adsorption tests were performed by using sample B′ as the selected adsorbent. The effects of solution pH, adsorption contact time, and interfering ions or ion selectivity diagrams on the uranium adsorption capacity are shown in Fig. 5. The uranium adsorption capacity as a function of the solution pH is shown in Fig. 5(a). The maximum uranium adsorption capacity of 207.595 mg/g was obtained at a solution pH of approximately 8, which can show that the synthesized MSs based on the phosphoric acid is one of the best candidates for uranium adsorption from seawater. As illustrated in Fig. 5(a), the uranium adsorption capacity on the synthesized MS increased dramatically at pH 1–8, and then decreased adsorption was observed at pH > 8. The dominant species of uranium ions at acidic pH is UO_2_^2+^ which have to compete with a high concentration of H^+^ and H_3_O^+^. In the higher pH, especially between 7–9, the uranium adsorption capacity increases greatly due to fewer H^+^ and H_3_O^+^ and higher adsorptive sites for uranium ions. At the pH > 9, The different anion species of uranium ions were generated such as UO_2_(OH)^3−^, UO_3_(OH)^7−^ which have to compete with the high concentration of OH^−^ and cause to decrease uranium adsorption capacity^47, 49, 52–54^. A fundamental issue in understanding the sorption mechanism of uranium on the synthesized MS samples in the equilibrium condition is studying their sorption isotherms. The adsorption isotherms for uranium on the sample B′ at optimum pH are shown in Fig. 5(b) and (c). The Langmuir and Freundlich models were used to simulate the experimental results. The Langmuir isotherm is defined based on the formation of a homogeneous monolayer adsorbate on the outer surface of the adsorbent without taking place any further adsorption. The Langmuir model can be expressed as:

3 $$\frac{{C}_{e}}{{q}_{e}}=\frac{{C}_{e}}{{q}_{m}}+\frac{1}{{q}_{m}{K}_{l}}$$

where *q*_*m*_ and *K*_*L*_ are the maximum adsorption capacity at equilibrium of the monolayer of the sorbent (*mg/g*), and the Langmuir adsorption constant (*L/mg*), respectively. The appropriate model for heterogeneous adsorption is usually Freundlich isotherm which is expressed as below:

4 $$\mathrm{ln}\,{q}_{e}=\,\mathrm{ln}\,{k}_{F}+\frac{1}{n}\,\mathrm{ln}\,{C}_{e}$$

where *k*_*F*_ and n are the Freundlich isotherm constant, and the dimensionless heterogeneity factor, respectively. Table 8 summarizes the resulted parameters and the correlation coefficient of the both models. As can be observed, the Langmuir adsorption isotherm shows the approximately 142.5 mg/g adsorption capacity with the low correlation coefficient of 0.7998. on the other hand, the exponential term value of Freundlich model i.e. the inverse of heterogeneity factor is less than unity which shows that this isotherm can be considered as favorable for uranium adsorption by synthesized mesoporous silica^1, 47, 55^. The effect of adsorption contact time of 5–60 min on the uranium adsorption capacity is shown in Fig. 5(d). As shown in this figure, sorption reaches equilibrium at its maximum value in the initial 10 min. These results indicated that the synthesized MSs exhibit considerably shorter time to reach its maximum value than that of previous reports. To clarify the sorption kinetics of uranium on synthesized MS sample and confirm measuring sorption capacity, the two important kinetics models including the pseudo-first- and second order kinetic models were applied to the experimental data (Fig. 5(e) and (f)). The pseudo-first- and second order kinetic models are explained by equations (5) and (6), respectively:

5 $$\mathrm{ln}({q}_{e}-{q}_{t})=\,\mathrm{ln}\,{q}_{e}-{k}_{1}t$$

6 $$\frac{t}{{q}_{t}}=\frac{t}{{q}_{e}}+\frac{1}{{q}_{e}^{2}{K}_{2}}$$

where *q*_*e*_ (*mg g* ^−*1*^) and *q*_*t*_ (*mg g* ^−*1*^) are the amounts of uranium adsorbed in synthesized MS sample (*mg g* ^−*1*^) at equilibrium and at time of *t* (*min*), respectively, and k_1_ (*min* ^−*1*^) and k_2_ (*g mg* ^−*1*^ *min* ^−*1*^) are the sorption rate constant of first and second-order kinetic model, respectively. The results show that the pseudo-second-order model realistically matches with the experimental kinetics data. This model gives a unity correlation coefficient and shows a much closer equilibrium capacity of 208.33 *mg/g* to the experimental value of 207.59 *mg/g*. The parameters of pseudo-first- and second order kinetic models are summarized in Table 9. The results suggest that more appropriate kinetic model to explain the kinetics of uranium sorption in synthesized MS sample is the pseudo-second-order model^55,56^. The significant achievement of the synthesized MSs based on the phosphoric acid is the selectivity of uranium adsorption. Figure 5(g) shows the uranium recovery percentages of sample B′ in the presence of other elements including Pb, Cu, Mo, Ni, K, and As. The obtained results indicated that the phosphoric-based MSs present the highest selectivity for uranium adsorption.

**Table 8:** The resulted parameters and the correlation coefficient of the Langmuir and Freundlich isotherms.

LANGMUIR isotherm	q_m_ (mg/g)	k_L_ (L/mg)	R^2^
	142.8571	2.5	0.7998
**FREUNDLICH isotherm**	**n**	**k**_**f**_	**R** ^**2**^
	7.849294	216.1225	0.9613

**Table 9:** The extracted parameters of pseudo-first- and second order kinetic models.

Kinetics model	Pseudo-first-order			Pseudo-second-order		
	q_e_ (mg/g)	k_1_ (min^−1^)	R²	q_e_ (mg/g)	k_2_ (g/mg min)	R²
	4.346103	0.028788	0.8146	208.333	26.041667	1

## Conclusions

Two kinds of MSs based on the acetic and phosphoric acids were synthesized by hydrothermal method for uranium [U(VI)] selective adsorption from aqueous solutions. The main idea of using phosphoric acids instead of acetic acid was the synthesis of enhanced PFG MS and consequently enhanced U(VI) adsorption. Different analysis methods including FT-IR, XRD, SEM, EDS, and BET were established to distinguish the structural characterization of synthesized samples. The uranium adsorption experiments were performed by batch method, and element concentrations were measured by ICP-OES. The XRD patterns and FT-IR spectra indicated that the synthesized MSs show the hexagonal structure (the p6m symmetry group) and the main absorption bands of MSs including ν_as_(Si–O–Si), ν(P=O), ν_s,as_(CH), and OH. The uranium adsorption for different synthesized MSs including 0, 3, 10, 20, and 100 phosphoric/acetic acid mass ratio indicated that the uranium adsorption capacity increases by increasing phosphoric acid mass ratio, e.g., from 55.75 mg/g to 207.6 mg/g for 0 and 100 mass ratios, respectively. The BET analysis indicated that the phosphoric acid-based MSs present extremely reduced specific surface area with respect to the acetic acid-based MSs. The SEM images also indicated that by the increasing phosphoric acid mass ratio, the morphologies of synthesized MSs extremely changed, which can be the main reason for reduced specific surface area. Meanwhile, EDS analysis shows that enhanced phosphorus can be the main reason to enhance PFGs and uranium adsorption. The increasing of PFGs can be related to branching out of each existing PFGs and producing chelating groups for selective uranium adsorption. The main achievement of this research is that the enhanced PFG MS presents the highest adsorption selectivity of 79.82% for U(VI) among six different elements including Pb, As, Cu, Mo, Ni, and K.
